# Mixed effects analysis of factors associated with health insurance coverage among women in sub-Saharan Africa

**DOI:** 10.1371/journal.pone.0248411

**Published:** 2021-03-19

**Authors:** Hubert Amu, Abdul-Aziz Seidu, Ebenezer Agbaglo, Robert Kokou Dowou, Edward Kwabena Ameyaw, Bright Opoku Ahinkorah, Kwaku Kissah-Korsah

**Affiliations:** 1 Department of Population and Behavioural Sciences, School of Public Health, University of Health and Allied Sciences, Hohoe, Ghana; 2 Department of Population and Health, University of Cape Coast, Cape Coast, Ghana; 3 College of Public Health, Medical and Veterinary Sciences, James Cook University, Townsville, Queensland, Australia; 4 Department of English, University of Cape Coast, Cape Coast, Ghana; 5 Department of Epidemiology and Biostatistics, School of Public Health, University of Health and Allied Sciences, Hohoe, Ghana; 6 School of Public Health, Faculty of Health, University of Technology Sydney, Sydney, Australia; National Cancer Center, Japan, JAPAN

## Abstract

**Introduction:**

In the pursuit of achieving the Sustainable Development Goal targets of universal health coverage and reducing maternal mortality, many countries in sub-Saharan Africa have implemented health insurance policies over the last two decades. Given that there is a paucity of empirical literature at the sub-regional level, we examined the prevalence and factors associated with health insurance coverage among women in in sub-Saharan Africa.

**Materials and methods:**

We analysed cross-sectional data of 307,611 reproductive-aged women from the most recent demographic and health surveys of 24 sub-Saharan African countries. Bivariable and multivariable analyses were performed using chi-square test of independence and multi-level logistic regression respectively. Results are presented as adjusted Odds Ratios (aOR) for the multilevel logistic regression analysis. Statistical significance was set at p<0.05.

**Results:**

The overall coverage of health insurance was 8.5%, with cross-country variations. The lowest coverage was recorded in Chad (0.9%) and the highest in Ghana (62.4%). Individual-level factors significantly associated with health insurance coverage included age, place of residence, level of formal education, frequency of reading newspaper/magazine and watching television. Wealth status and place of residence were the contextual factors significantly associated with health insurance coverage. Women with no formal education were 78% less likely to be covered by health insurance (aOR = 0.22, 95% CI = 0.21–0.24), compared with those who had higher education. Urban women, however, had higher odds of being covered by health insurance, compared with those in the rural areas [aOR = 1.20, 95%CI = 1.15–1.25].

**Conclusion:**

We found an overall relatively low prevalence of health insurance coverage among women of reproductive age in sub-Saharan Africa. As sub-Saharan African countries work toward achieving the Sustainable Development Goal targets of universal health coverage and lowering maternal mortality to less than 70 deaths per 100,000 live births, it is important that countries with low coverage of health insurance among women of reproductive age integrate measures such as free maternal healthcare into their respective development plans. Interventions aimed at expanding health insurance coverage should be directed at younger women of reproductive age, rural women, and women who do not read newspapers/magazines or watch television.

## Introduction

Achievement of universal health coverage (UHC) and reduction of maternal mortality are major targets of the Sustainable Development Goal (SDG) Three, which seeks to promote health for all at all ages by the year 2030 [1]. Reduction of maternal mortality is also a proxy for assessing improvement in maternal health in the scope of the SDGs. Health insurance is a key driver of UHC, as it serves as a financial risk protection mechanism which provides affordable health coverage for the indigenes in low- and middle-income countries (LMICs) [2, 3]. Protecting women from the financial predicament of out-of-pocket payments for healthcare through health insurance coverage ensures that they have access to the appropriate orthodox healthcare especially during pregnancy and childbirth which are responsible for a significant proportion of the mortality occurring among them in LMICs [4].

In the pursuit of achieving the SDG target of UHC, many countries in sub-Saharan Africa (SSA) have implemented health insurance policies over the last two decades [4]. Many of the schemes currently cover public servants at both central and local government levels together with their family members while others cover the entire population [5–14]. The health insurance schemes in SSA have been introduced to largely replace the existing health financing systems which are dominated by out-of-pocket payments. Health insurance has, thus improved access to quality healthcare services and provided financial risk protection for the populace with special focus on the vulnerable including women of reproductive age [15–18].

While there is a paucity of the empirical literature on the factors influencing health insurance coverage at the sub-regional level, studies conducted at individual country levels and among up to five sub-Saharan African countries (Ghana, Kenya, Nigeria, Tanzania, & Uganda) have shown that socio-demographic factors such as occupation, wealth status, residence, level of education, and age significantly influence coverage of health insurance [19–24]. To provide empirical literature and bridge the existing knowledge gap for the generality of SSA, we analysed the prevalence and predictors of health insurance coverage from 24 in sub-Saharan African countries, using nationality representative demographic and health survey (DHS) data. We adopted multilevel logistic regression to achieve a robust mixed effects analysis of the individual and contextual factors influencing the coverage of health insurance in SSA. Our findings could inform policymakers of the factors to consider when planning and implementing health insurance-related interventions geared towards achieving UHC and reducing maternal mortality.

## Materials and methods

### Data source

Data for this study were obtained from Demographic and Health Surveys (DHS) conducted between January 1, 2010, and December 31, 2019, in 24 sub-Saharan African countries (see Table 1). Specifically the womens’ files of the DHS were used. The choice of the 24 countries was influenced by the availability of the variables of interest in their datasets. DHS is a nationwide survey undertaken across LMICs every five years [25]. The survey is representative of each of these countries and targets core maternal and child health indicators such as health insurance coverage. In selecting the sample for each survey in the various countries, a multi-stage sampling approach was employed. The first step of this sampling approach involved the selection of clusters (i.e., enumeration areas [EAs]), followed by systematic household sampling within the selected EAs. In this study, the sample size consisted of women aged 15–49 who had complete cases on all the variables of interest (N = 307,611). We followed the ‘Strengthening the Reporting of Observational Studies in Epidemiology’ (STROBE) statement in conducting this study and writing the manuscript (see S1) [26]. The data underlying the results presented in the study are available from https://dhsprogram.com/data/available-datasets.cfm.

**Table 1:** Description of the sample size.

Country	Year	Weighted Sample	Weighted Percentage
1. Benin	2017–2018	15,410	5.0
2. Burundi	2016–2017	16,783	5.5
3. * Congo DR	2013–2014	18,667	6.1
4. Ethiopia	2016	15,299	5.0
5. Gabon	2012	8,213	2.7
6. Ghana	2014	9,365	3.0
7. Gambia	2013	10,051	3.3
8. Guinea	2018	10,553	3.4
9. Kenya	2014	14,501	4.7
10. Liberia	2013	9,013	2.9
11. Lesotho	2014	2,849	0.9
12. Mali	2018	10,410	3.4
13. Malawi	2015–2016	24,540	8.0
14. Nigeria	2018	28,582	9.3
15. Niger	2012	11,023	3.6
16. Namibia	2013	9,100	3.0
17. Sierra Leone	2013	16,350	5.3
18. Chad	2014–2015	5,940	1.9
19. Togo	2013–2014	9,381	3.1
20. Tanzania	2015–2016	13,253	4.3
21. Uganda	2016	18,458	6.0
22. South Africa	2016	4,049	1.3
23. Zambia	2018	16,014	5.2
24. Zimbabwe	2015	9,809	3.2
All Countries	-	307,611	100

*DR = Democratic Republic

### Study variables

The outcome variable in this study was health insurance coverage. This was derived from the question ‘Are you covered by any health insurance?’ It was coded 1 = “Yes” and 0 = “No” [19, 27]. Both individual and contextual (household and community level factors) level factors were considered as explanatory variables in this study. The individual-level factors were age, marital status, educational level, employment, parity, and exposure to the mass media (radio, newspaper and television). The contextual variables were sex of household head, household wealth quintile, place of residence and sub-region (see Table 2). In the DHS, wealth was computed using data on household ownership of selected assets such as bicycle, materials used for house construction, television, type of water access and sanitation facilities were used. Wealth quintile was then created from these assets through Principal Component Analysis (PCA) by placing households on a continuous measure of relative wealth after which households were grouped into five wealth quintiles namely poorest, poorer, middle, richer and richest [25]. The sub-region variable was derived by aggregating countries based on their geographical location on the African continent (thus Western, Southern, Eastern, and Central). Our choice of the explanatory variables were influenced by their inclusion in the DHS datasets and previous literature which found these variables to be associated with health insurance coverage [5, 19–24, 27].

**Table 2:** Socio-demographic characteristics and health insurance coverage among women in in sub-Saharan Africa.

Variables	Weighted (N = 307,611)		Health insurance coverage		
	n	%	No (%)	Yes (%)	P-values
**Individual-level factors**					
** Age**					p<0.001
** **15–19	61,599	20.0	93.3	6.9	
** **20–24	55,777	18.1	93.3	6.7	
** **25–29	54,677	17.8	91.7	8.3	
** **30–34	45,511	14.8	90.2	9.8	
** **35–39	38,719	12.6	89.6	10.4	
** **40–44	28,223	9.2	88.8	11.3	
** **45–49	23,106	7.5	89.1	10.9	
**Marital status**					p<0.001
** **Never married	80,822	26.3	90.8	9.2	
** **Married	168,425	54.8	92.1	7.9	
Cohabiting	30,783	10.0	87.8	12.2	
Widowed	8,444	2.8	91.9	8.1	
Divorced	19,136	6.2	92.5	7.6	
**Educational level**					p<0.001
** **No education	92,888	30.2	96.1	3.9	
** **Primary	99,495	32.3	93.6	6.4	
** **Secondary	98,832	32.1	87.3	12.7	
** **Higher	16,396	5.3	71.9	28.1	
**Employment**					p<0.001
Not working	100,209	32.6	92.5	7.5	
Managerial	14,048	4.6	70.6	29.4	
Sales	56,511	18.4	92.0	8.0	
House/domestic	6,601	2.2	89.9	10.1	
Agricultural	78,344	25.5	92.7	7.3	
Services	22,872	7.4	92.7	7.3	
Manual	26,036	8.5	93.6	6.5	
Clerical	2,989	1.0	72.4	27.6	
**Parity**					p<0.001
** **1–3 children	120,208	39.1	90.3	9.7	
** **4+	108,687	35.3	92.5	7.5	
** **None	78,716	25.6	91.5	8.7	
**Frequency of listening to radio**					p<0.001
Not at all	119,195	38.8	94.2	5.8	
** **Less than once a week	65,306	21.2	90.9	9.1	
** **At least once a week	113,563	36.9	88.9	11.2	
Almost every day	9,548	3.1	87.0	12.9	
**Frequency of reading newspaper or magazine**					p<0.001
** **Not at all	238,357	77.5	93.3	6.7	
** **Less than once a week	37,626	12.2	87.1	12.9	
** **At least once a week	29,126	9.5	80.6	19.4	
Almost every day	2,503	0.8	74.9	25.1	
**Frequency of watching television**					p<0.001
** **Not at all	180,039	58.5	95.3	4.7	
** **Less than once a week	41,284	13.4	90.6	9.4	
** **At least once a week	72,048	23.4	84.6	15.4	
Almost every day	14,240	4.6	72.8	27.2	
**Contextual factors**					
**Sex of household head**					p<0.001
** **Male	221,333	72.0	91.9	8.1	
** **Female	86,278	28.1	90.1	9.9	
** Wealth quintile**					p<0.001
** **Richest	74,021	24.1	85.1	15.0	
** **Richer	64,330	20.9	91.9	8.1	
** **Middle	59,132	19.2	93.7	6.3	
** **Poorer	56,717	18.4	94.4	5.6	
** **Poorest	53,412	17.4	92.9	7.1	
**Place of residence**					p<0.001
** **Urban	116,585	37.9	86.9	13.1	
** **Rural	191,026	62.1	94.0	6.0	

### Statistical analyses

The data were analysed with Stata version 14.2 for macOS (Stata Corporation, College Station, TX, USA). Three steps were followed to analyse the data. The first step was the use of descriptive statistics to describe the sample and cross-tabulation of all the explanatory variables against health insurance coverage. The second step was a bivariable analysis to select potential variables for the regression analysis. Variables that were statistically significant at the bivariable analysis stage at p<0.05 were moved to the final step, where two levels of multilevel logistic regression models were built to assess the individual and contextual factors associated with health insurance coverage.

Clusters were considered as random effects to account for the unexplained variability at the contextual level [28]. We fitted four models (see Table 3). The first model was the empty model (Model I), which showed the variation in health insurance coverage attributed to the distribution of the primary sampling units (PSUs) in the absence of the explanatory variables. Model II had only the individual level factors and health insurance coverage. The purpose of Model II was to look at how the individual level factors are associated with health insurance coverage in the absence of the contextual factors. Model III was developed to look at the association between the contextual level factors and health insurance coverage, in the absence of the individual level factors. The final model (Model IV) was the complete model that had the individual and contextual level factors and health insurance. The purpose was to look at the association between both the individual and contextual level factors and health insurance coverage. For all the models, adjusted Odds Ratios (aOR) and their associated 95% confidence intervals were presented. These models were fitted by the Stata MLwinN software version 3.05 [29].

**Table 3:** Multilevel logistic regression of individual and contextual factors associated with health insurance coverage among women in sub-Saharan Africa.

Variables	Model I	Model II AOR [95%CI]	Model III AOR [95%CI]	Model IV AOR [95%CI]
***Fixed effects***				
**Individual-level factors**				
**Age**				
15–19		0.37***[0.34,0.40]		0.36***[0.33,0.38]
20–24		0.34***[0.32,0.36]		0.33***[0.31,0.35]
25–29		0.48***[0.45,0.51]		0.47***[0.44,0.50]
30–34		0.66***[0.62,0.70]		0.66***[0.62,0.70]
35–39		0.80***[0.75,0.85]		0.79***[0.75,0.84]
40–44		0.94*[0.88,0.99]		0.94* [0.88,0.99]
45–49		Ref		Ref
**Marital status**				
Never married		Ref		Ref
** **Married		0.94***[0.89,0.98]		0.94**[0.89,0.98]
Cohabiting		1.37***[1.30,1.45]		1.22***[1.15,1.30]
Divorced		0.73***[0.67,0.81]		0.69***[0.63,0.76]
Widowed		0.70***[0.65,0.75]		0.65***[0.60,0.69]
**Educational level**				
No education		0.24***[0.22,0.26]		0.22***[0.21,0.24]
Primary		0.40***[0.37,0.43]		0.39***[0.36,0.41]
Secondary		0.69***[0.65,0.73]		0.68***[0.65,0.72]
Higher		Ref		Ref
**Employment**				
Not working		Ref		Ref
Managerial		2.22***[2.10,2.34]		2.12*** [2.00,2.24]
Sales		2.03*** [1.84,2.23]		2.07***[1.87,2.28]
House/domestic		0.95***[0.91,0.99]		0.91***[0.87,0.96]
Agricultural		1.14**[1.04,1.25]		1.06 [0.96,1.17]
Services		1.67***[1.61,1.74]		1.67***[1.60,1.75]
Manual		0.82***[0.77,0.87]		0.77***[0.72,0.81]
Clerical		0.88***[0.83,0.94]		0.85***[0.82,0.98]
**Parity**				
1–3		1.01[0.96,1.05]		1.04[0.98,1.09]
4 and above		0.84***[0.80,0.88]		0.82***[0.78,0.87]
None		Ref		Ref
**Frequency of listening to radio**				
Not at all		0.84***[0.81,0.87]		0.81***[0.77,0.84]
Less than once a week		1.01[0.97,1.05]		0.99 [0.95,1.03]
At least once a week		Ref		Ref
Almost every day		0.52***[0.48,0.57]		0.73***[0.66,0.80]
**Frequency of reading newspaper or magazine**				
Not at all		0.83***[0.80,0.87]		0.80***[0.76,0.84]
Less than once a week		0.82**[0.78,0.86]		0.84*** [0.81,0.86]
At least once a week		Ref		Ref
Almost every day		0.98[0.86,1.10]		1.12[0.99,1.28]
**Frequency of watching television**				
Not at all		0.42***[0.40,0.43]		0.41***[0.40,0.43]
Less than once a week		0.71***[0.68,0.73]		0.71***[0.26,0.30]
At least once a week		Ref		Ref
Almost every day		2.56***[2.41, 2.72]		3.00***[2.81,3.21]
**Contextual factors**				
**Sex of household head**				
Male			0.84***[0.81,0.86]	0.92**[0.88,0.98]
Female			Ref	Ref
**Wealth status**				
Richest			0.64***[0.61,0.68]	1.61***[1.51,1.71]
Richer			0.50***[0.47,0.52]	0.99 [0.94,1.06]
Middle			0.54***[0.52,0.57]	1.00[0.95,1.06]
Poorer			0.60***[0.57,0.62]	0.92***[0.88,0.96]
Poorest			Ref	Ref
**Place of residence**				
Rural			Ref	Ref
Urban			1.73***[1.67,1.80]	1.20***[1.15,1.25]
**Sub-region**				
Southern			0.66***[0.61,0.72]	0.25***[0.23,0.27]
Central			Ref	Ref
Eastern			1.74***[1.67,1.82]	1.09***[1.04,1.14]
Western			1.27***[1.22,1.32]	1.07**[1.03,1.12]
***Random effects***				
**Contextual level**				
Variance (SE)	0.315[0.282–0.349]	0.364[0.326–0.402]	0.317[0.283–0.350]	0.336[0.301–0.372]
ICC (%)	16.0[13.9–18.7]	19.2[17.0–25.8]	16.4[13.7–26.2]	16.0[13.4–17.4]
MOR	1.71[1.66–1.76]	1.78[1.72–1.83]	1.71[1.66–1.76]	1.74[1.69–1.79]
*N*	307,611	307,611	307,611	307,611

Exponentiated coefficients; 95% confidence intervals in brackets

* p < 0.05

** p < 0.01

*** p < 0.001

SE = Standard Error; ICC = Intra-Class Correlation; LR Test = Likelihood ratio Test; MOR: Median Odds Ratio

Model I is the null model, a baseline model without any determinant variable

Model II = individual-level variables

Model III = contextual variables

Model IV is the final model adjusted for individual and contextual variables

Using the variance inflation factor (VIF), the multicollinearity test showed that there was no evidence of collinearity among the explanatory variables (Mean VIF = 1.54, Maximum VIF = 2.09 and Minimum VIF = 1.09). The choice of reference categories were informed by the categories with lower likelihood of using NHIS. For example, in the case of age, those aged 15–19 were chosen as reference category. Where this could not be determined per literature, the category with the greatest number of observations was taken as a reference category. According to Hatt and Waters [30], pooling data can reveal broader results that are ‘‘often obscured by the noise of individual data sets.” To calculate the pooled values an additional adjustment is needed to account for the variability in the number of individuals sampled in each country. This is accomplished using the weighting factor 1/(A*n_c_/n_t_), where A is the number of countries asked a particular question, n_c_ is the number of respondents for the country c, and n_t_ is the total number of respondents over all countries asked the question [31].

### Ethical clearance

The DHS receive ethical clearance from the Ethics Review Committee of ORC Macro Inc. and the Ethics Review Committees of partner organizations in the various countries where the surveys are conducted.

## Results

### Prevalence of health insurance coverage

**Fig 1:**
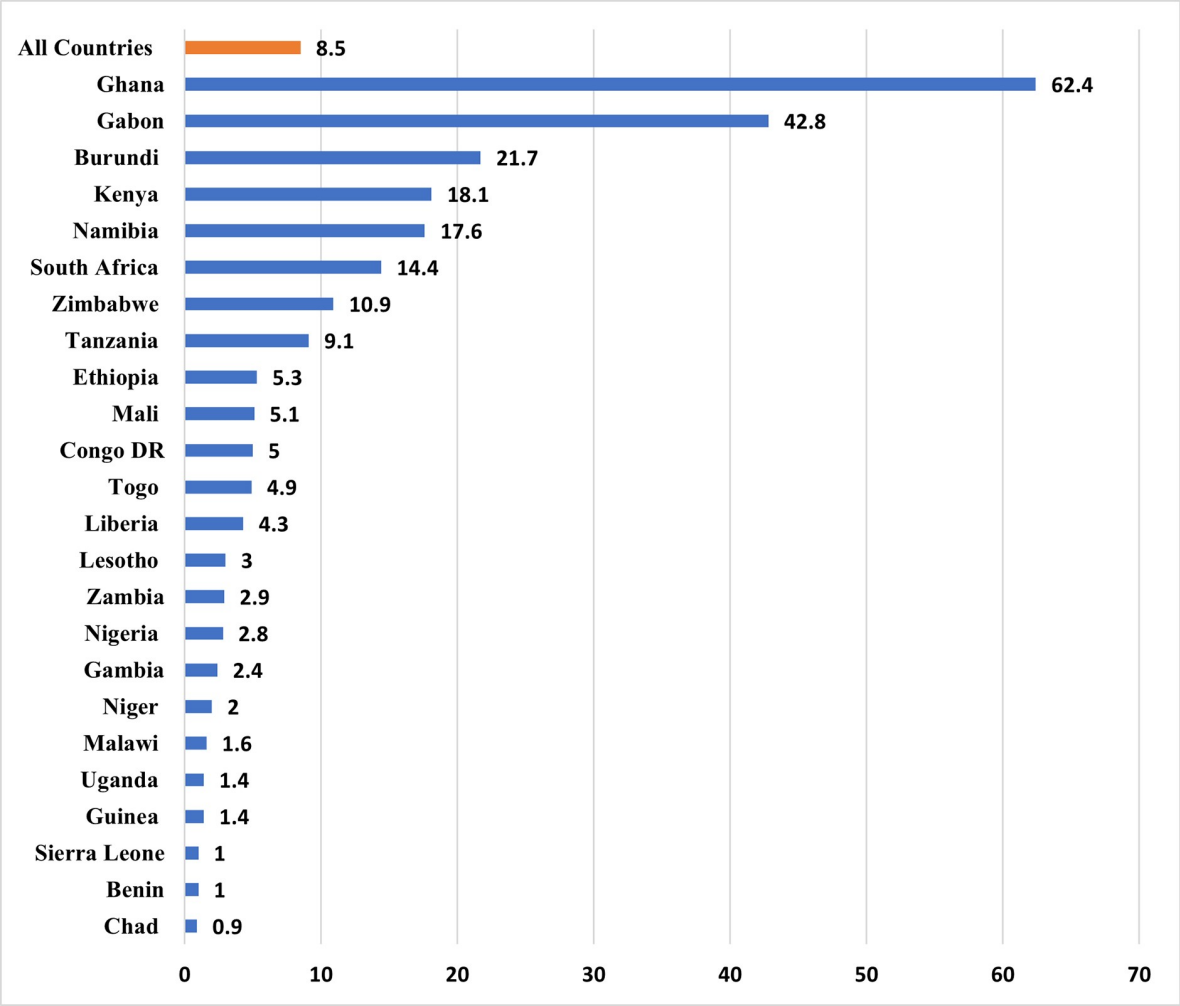
Prevalence (percentage) of health insurance coverage among women in in sub-Saharan Africa.

The prevalence of health insurance coverage among women in SSA is presented in Fig 1. The study showed that 8.5% of women in SSA are covered by health insurance, ranging from 0.9% in Chad to 62.4% in Ghana.

Table 2 presents the results on the socio-demographic characteristics and health insurance coverage among women in SSA. Twenty per cent of the women were aged 15–19, 54.8% were married, 32.3% had primary level of formal education, 32.6% were not working and 39.1% had parity 1–3. With mass media exposure, it was found that 38.8%, 77.5% and 58.5% had not listened to radio, not read a newspaper or magazine and did not watch television at all respectively. The study further revealed that 91.9% were in male-headed households. About 6 out of every 10 women lived in rural areas. About 24% of the women were in the richest wealth quintile. The chi-square analysis showed that all the explanatory variables demonstrated statistically significant associations with health insurance coverage among women in SSA.

### Multilevel logistic regression of the factors associated with health insurance coverage among women in sub-Saharan Africa

Table 3 shows the multilevel logistic regression results of individual and contextual factors associated with health insurance coverage among women in SSA. Compared to those aged 45–49, women in the various age groups had lower odds of being covered by health insurance. The study showed that cohabiting women [aOR = 1.22, 95% CI = 1.15–1.30] were more likely to be covered by health insurance compared women who had never married. Compared with women who had higher education, those with no education [aOR = 0.22, 95% CI = 0.21–0.24] had the lowest odds of health insurance coverage.

With employment, compared with those who do not work, those in managerial roles [aOR = 2.12, 95% CI = 2.00–2.24] and those into sales [aOR = 2.07, 95% CI = 0.45–0.54] were more likely to be covered by health insurance. On the contrary, clerics [aOR = 0.88, 95% CI = 0.83–0.94] and women into manual occupation were less likely to be covered by health insurance. Women with parity 4 and above [aOR = 0.82, 95% CI = 0.78–0.87] were less likely to be covered by health insurance than women at parity zero. Women who did not listen to radio at all [aOR = 0.81, 95% CI = 0.77–0.84] and those who listened to radio almost everyday [aOR = 0.73, 95% CI = 0.66–0.80] had lower odds of health insurance coverage compared with those who listened to radio at least once a week. Regarding the frequency of reading newspaper or magazine, those who did not read newspaper or magazine at all [aOR = 0.80, 95% CI = 0.76–0.84] and those who read less than once a week [aOR = 0.84, 95% CI = 0.81–0.86] had lower odds of health insurance coverage compared with those who read at least once a week.

Those who did not watch television at all [aOR = 0.41, 95% CI = 0.40–0.43] had the lowest odds of health insurance coverage compared with those who watched at least once a week. Women in male-headed households [aOR = 0.92, 95% CI = 0.88–0.98] were less likely to be covered by NHIS compared with those in female-headed households. We also found that compared with those in the poorest wealth quintile, women in richest wealth quintile had higher odds of being covered by health insurance [aOR = 1.61, 95% CI = 1.51–1.71]. Those in the urban areas had higher odds of being covered by health insurance, compared with those in the rural areas [aOR = 1.20, 95% CI = 1.15–1.25]. Compared with Central Africa, women from Eastern [aOR = 1.09, 95% CI = 1.04–1.14] and Western [aOR = 1.07, 95% CI = 1.03–1.12] Africa were more likely to be covered by health insurance.

## Discussion

We examined the prevalence and determinants of health insurance coverage among women in 24 sub-Saharan African countries. Our results showed an overall low coverage of health insurance (8.5%) among women in SSA, with cross-country variations where the lowest prevalence was recorded in Chad (0.9%) and the highest in Ghana (62.4%). In many of the countries where health insurance coverage is quite high, women have been prioritised under the health insurance policies of such countries. These include free maternal health policies embedded into the health insurance schemes and exemptions from the payment of annual premiums during pregnancy and childbirth. These interventions make antenatal and skilled delivery services free for women [32–34].

Our findings where women in other age brackets recorded lower odds of health insurance coverage confirm the observations of Ibok [35] in Nigeria, Mhere [36] in Zimbabwe, Kimani [37] in Kenya, Mulenga et al. [38] in Zambia, and Duku [39] in Ghana. Several explanations have been given for this association. Mhere [36] posited that as people grow older, they develop a greater sense of purpose for life. This comes with a sense of responsibility, including the need to take better care of one’s health. Kimani et al. [37] also explained this association within the context of the direct link between increases in age and increase in financial security. Thus, as people grow and become financially stable, they are able to cater for the costs involved in enrolling on health insurance schemes.

Married and cohabiting women having higher of health insurance coverage in our study confirms similar findings that have been made in in Nigeria [35], Zambia [38], Kenya [37], Ghana [39], Russia [40], USA [41], and Jamaica [42]. In many of the sub-Saharan African countries where parental health insurance coverage automatically covers children, the higher probability of married/cohabitting women to subscribe to health insurance may be due to their decision to use health insurance to mitigate personal and child healthcare financial burden [39]. It is also possible that married and cohabiting women would get some support and encouragement from their partners to help them subscribe to health insurance [38]. Married/cohabiting women are also likely to benefit from the financial advantages of living in dual-income households, including better opportunities to healethcare access [37].

In our study, non-educated women were less likely to be covered by health insurance compared with educated ones. This finding is congruent to the findings of some previous studies [35, 37–44]. Our finding could be due to the fact that compared with educated women, non-educated women may be more likely to subscribe to health insurance as a way of avoiding expenditure that may come with some eventual unforeseen health issues [38]. Additionally, higher educational attainment is likely to predispose the individual to various kinds of information, including the need to take good care of one’s health. Uneducated women, on the other hand, may be less likely to have access to such information. This could explain the lower likelihood of health insurance subscription among women without formal education.

We found that reproductive age women in urban areas had higher odds of being covered by health insurance. This finding reflects the persistent challenges of social inequalities in health across SSA [45–49]. While health facilities are for instance, mostly cited in urban areas, health professionals also refuse posting to rural areas. Access to healthcare is thus, usually a challenge for reproductive age women especially in maternal (pregnancy and childbirth) situations. As health insurance schemes constitute a part of the healthcare system which are mostly focused on urban areas, it is, therefore, not surprising that the probability of coverage was lower in rural areas.

Our findings where women in managerial work recorded higher likelihoods of health insurance coverage could mean that formal employment, such as managerial work, gives individuals financial autonomy that can help them take care of the bills that come with health insurance, and thus result in a higher likelihood of health insurance. This finding is also consistent with the findings of some previous studies [35, 37, 40]. Our study recorded lower odds of health insurance coverage of wealthier women, compared with those from the poorest wealth quantile. Wealthier women are likely to use private health facilities, due to the quality of healthcare provided [40] while in most sub-Saharan African countries, social health insurance is usually applicable to public health facilities [44]. Other studies, however, recorded higher odds of health insurance subscription among women from wealthier households [37, 38, 50]. This may explain why we found that women in the richest households to be covered by health insurance compared to poorest women in our multi-level regression Model III which was a bivariable analysis.

Our results revealed a significant association between mass media exposure and health insurance coverage. We found lower likelihoods of health insurance coverage among mothers who were not exposed to mass media (radio, TV, and newspapers), compared with those who use mass media frequently. This finding is consistent with findings of previous studies that reported a significant association between mass media exposure and health insurance coverage [35, 37, 38]. Users of mass media are privy to information on a wide variety of issues, including information on health-related issues, such as health insurance. Thus, women who do not use mass media may miss some relevant information that could have encouraged them to use health insurance, and this could be a reason for the lower likelihood of health insurance usage among women who were not exposed to mass media. Research has also revealed the effectiveness of using mass media for health promotion programs intended to increase public awareness on health issues such as health insurance [51].

### Strengths and limitations

A key strength of our study is that we used nationally representative data of the various countries for the analysis. This, coupled with the multi-country nature of the study, address the issue of generalisability of our findings. We also used the current versions of the DHS data of the respective countries. This ensures that our findings reflect the current realities of the health insurance situation. Additionally, the data collection of the surveys used for the studies featured high standard methodological procedures, as well as highly experienced field assistants and these guarantee high quality of the data used. Moreover, we used higher-order statistics, such as multi-level binary logistic regression, for our analysis. This ensured a robust analysis of the data and catered for issues of validity.

Despite the strengths of this study, the findings need to be interpreted with caution, given that it is characterised by certain limitations. It is important to note, for instance, that the cross-sectional design adopted in collecting the DHS data limits our ability to make any causal inferences among the variables studied. Besides, the study possibly suffers from recall bias that often characterises the DHS, given the retrospective nature of reporting health insurance coverage among the study participants in the respective countries.

## Conclusion

Generally, we found a relatively low prevalence of health insurance coverage among reproductive age women in SSA. As SSA countries move towards achieving the SDG targets of achieving universal health coverage and reducing maternal mortality to less than 70 deaths per 100,000 live births, it is imperative for sub-Saharan African countries with a low prevalence of health insurance coverage among women in reproductive age to integrate interventions like free maternal healthcare into their respective health insurance packages. Younger reproductive age younger women, those in rural areas, divorced, the uneducated, those who do not read newspapers/magazines or watch television, and the rich. The mass media, in particular, can be used for health insurance promotion programs among women in reproductive age, given its effectiveness in increasing people’s awareness of health issues.
